# Development of an improved and accessible diet for western corn rootworm larvae using response surface modeling

**DOI:** 10.1038/s41598-019-52484-z

**Published:** 2019-11-05

**Authors:** Man P. Huynh, Bruce E. Hibbard, Michael Vella, Stephen L. Lapointe, Randall P. Niedz, Kent S. Shelby, Thomas A. Coudron

**Affiliations:** 1 Division of Plant Sciences, University of Missouri, Columbia, Missouri 65211 USA; 2 Plant Genetics Research Unit, USDA-Agricultural Research Service, Columbia, Missouri 65211 USA; 3 Frontier Scientific Services, Newark, Delaware, 19711 USA; 4 United States Horticultural Research Laboratory, USDA-Agricultural Research Service, Fort Pierce, Florida 34945 USA; 5 Biological Control of Insects Research Laboratory, USDA-Agricultural Research Service, Columbia, Missouri 65203 USA

**Keywords:** Assay systems, Entomology

## Abstract

The western corn rootworm (WCR), *Diabrotica virgifera virgifera* LeConte, is an important pest of maize (*Zea mays* L.). Published WCR diets contain corn root powder, which is not available for purchase, thereby limiting the practical use of diets containing this ingredient. We applied response surface modeling combined with mixture designs to formulate a WCR diet that does not require corn root powder. We developed the new formulation by systematically exploring eight protein ingredients from animal, plant, and yeast sources based on simultaneous evaluation of three life history parameters (weight, molting, and survival). This formulation (WCRMO-2) without corn root powder supported approximately 97% of larval survival and successful molting. Larval weight gain after 10 days of feeding on WCRMO-2 was 4-fold greater than that of larvae feeding on the current best published WCR diet. Additionally, there was no significant difference in these larval performance traits when larvae were reared on WCRMO-2 and the best proprietary WCR diet. A commercial version of WCRMO-2 was tested and found to perform comparably for these traits. These improvements met our goal of a diet comprised of available ingredients that supports performance of WCR larvae equal to or better than publicly available formulations and proprietary formulations.

## Introduction

The western corn rootworm (WCR), *Diabrotica virgifera virgifera* LeConte, is an economically important pest of maize (*Zea mays* L.) in North America and parts of Europe. The impact of corn rootworm has been estimated at approximately $2 billion annually^1^. Yield reduction by this pest primarily results from larval feeding on maize roots^2,3^ that causes detrimental effects on nutrient and water uptake^4^, facilitation of pathogen infestation^5,6^, and unharvested grain due to lodging^2,3,7^. A variety of management tactics [e.g. crop rotation, chemical insecticides, transgenic maize expressing insecticidal proteins from *Bacillus thuringiensis* Berliner (Bt)] have faltered due to adaptation of WCR populations^8–15^. Insect resistance management (IRM) plans for western corn rootworm have been directed toward monitoring the development of resistance to each of the Bt products^16^. Diet bioassays can be a key component of IRM plans. An ideal diet^17^ used in diet bioassays should be publicly available, compatible with insecticide toxins, easy to use, low in contamination, and support larval development similar to larvae feeding on corn roots.

We previously developed an improved diet for WCR larvae, hereafter referred to as “WCRMO-1”^18^, an optimization of the ingredients in a previously published diet for WCR. Pleau *et al*.^19^ developed the first WCR diet, which was modified from a diet for southern corn rootworm (SCR), *D*. *undecimpunctata howardi* Barber^20–22^, by adding corn root powder, removing formalin, optimizing the pH and changing the concentrations of wheat germ and linseed oils. These modifications resulted in a diet that doubled larval weight gain compared to larvae reared on the SCR formulation^19^. We improved the Pleau *et al*. diet by optimizing the concentration of several ingredients (i.e., agar, casein, cellulose, corn root powder, linseed oil, and sucrose) and by adding wheat germ oil^18^. This resulted in a formulation that supported approximately 99% of larvae for survival and molting and further doubled the weight gain after 11 days compared with larvae reared on the Pleau *et al*. diet. Additionally, this formulation is publicly available, compatible with each of the four marketed Bt toxins^23^ and, under our laboratory conditions, resulted in fewer than 1% of infested diet wells becoming contaminated^18,24^.

Mixture designs combined with response surface modeling have been highly efficient for use in diet improvement for WCR^18^, northern corn rootworm, *D*. *barberi* Smith & Lawrence^25^, and another coleopteran species, *Diaprepes abbreviatus* (Linnaeus)^26,27^. Since insect diets are a mixture of several ingredients, a change in the number of ingredients and in the proportion of any ingredient results in a change in the relative composition of all ingredients. Mixture experiments are commonly analyzed based on Scheffé polynomials^28^ wherein the proportions of mixture components are varied and total amount of the mixture remains constant. Later, Piepel and Cornell^29^ developed a mixture-amount experiment expressing the parameters of the Scheffé model while varying the total amount of the mixture. The application of mixture experiments in combination with response surface modeling allows concurrently varying multiple ingredients to identify and characterize key components and predict an optimum formulation that maximizes all desired developmental traits^17,18,27,30,31^.

Previous diet work for WCR highlighted an important role of corn root powder as a key component that had positive effects on WCR development. Exclusion of this ingredient resulted in a slower development of WCR^18,19,32^. Corn root powder is the only ingredient that is not available for commercial purchase, thus limiting its use. In order to develop an ideal diet for WCR, this study focused on the development of a WCR diet with improved larval development and without corn root powder, making the WCR diet more widely available for researchers. By applying mixture designs combined with response surface modeling, we explored eight different protein ingredients, which are referred to diet ingredients that contain proteins as major components and may contain other nutritional components, derived from animal, plant, and yeast sources to identify key proteins. We then optimized proteins blends based on WCR life history parameters (weight, molting, and survival) while limiting contamination.

## Results

### Eight-protein screening experiment

**Figure 1:**
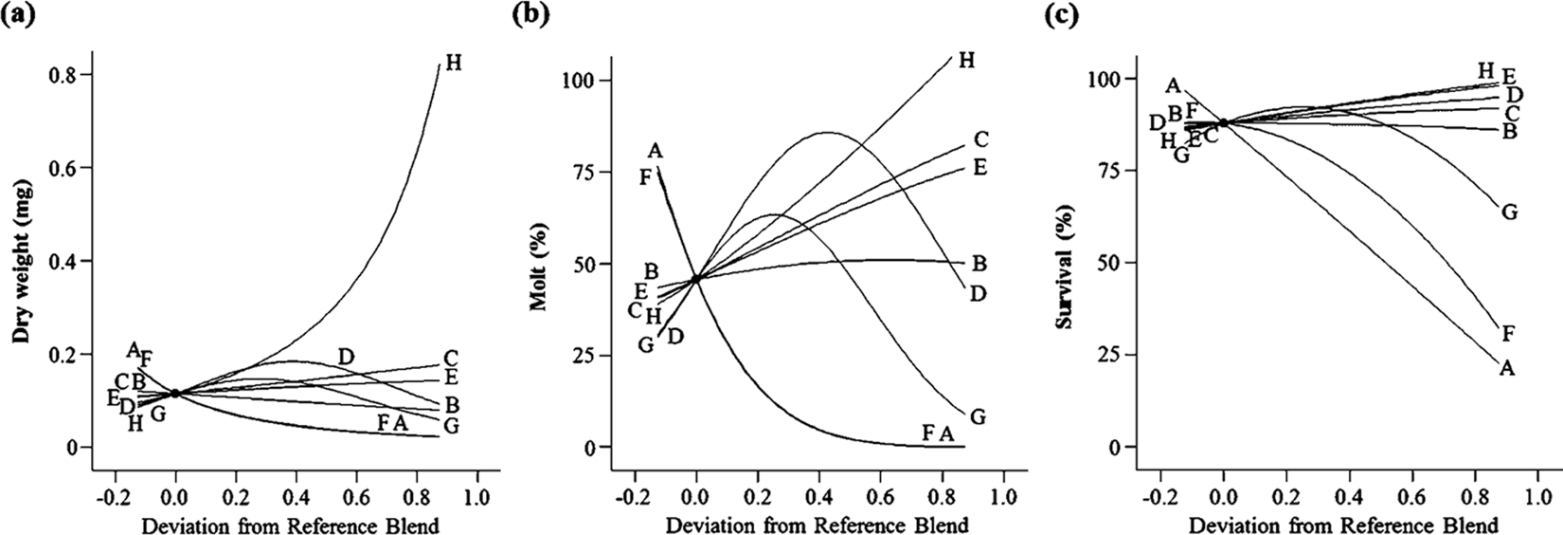
Trace plot of WCR larval responses deviation from a reference blend diet in 8-protein screening experiment. (**a**) weight, (**b**) molting, (**c**) survival. Reference blend proportions: corn gluten meal = cottonseed meal = casein = plant protein = whey protein = perfect amino = yeast extract = egg powder = 0.375. A: corn gluten meal, B: cottonseed meal, C: casein, D: plant protein, E: whey protein, F: perfect amino, G: yeast extract, H: egg powder.

The eight-protein mixture experiment produced significant response surface models for all three measures of life history parameters including weight (*p < *0.0001, *F*_*9*,*20*_* = *42.26) with insignificant lack of fit (LOF) (*p = *0.5382), molting (*p < *0.0001, *F*_*11*,*18*_ = 197.99) with significant LOF (*p = *0.0397), and survival (*p < *0.0001, *F*_*9*,*20*_ = 50.80) with significant LOF (*p = *0.0009) by varying 8 different protein sources: corn gluten meal, cottonseed meal, casein, plant protein, whey protein, perfect amino, yeast extract, and egg powder (see Supplementary Table S4). Pure errors of models for molting (sum of square (SS) of pure error = 0.013) and survival (SS of pure error = 0.0003) were very small, and resulted in significant LOFs. All models had R-squared, predicted R-squared and adjusted R-squared values in reasonable agreement, i.e. the differences between the predicted and adjusted R-squared values were <0.2, indicating good predicted models. The relationships between protein sources and larval performance were shown in trace plots that determine the effects of changeable proportions of one component in relation to a reference blend while all the relative proportions of all other components were held constant^30,33^ (Fig. 1). The direction and magnitude of influence of the individual components on the measured response variables are indicated by the slope of the line.

All response models revealed that the three components responsible for the greatest effects on all overall larval developmental traits (weight, molting, and survival) were three animal protein sources: casein, egg powder, and whey protein (Fig. 1a–c). For plant protein sources, plant protein and cottonseed meal, to some extent, had positive effects on all three criteria of larval performance, whereas corn gluten meal and Perfect Amino® had negative effects on all response measures. Yeast extract had minor positive effects on all larval performance compared to other positive components tested.

### Three-protein mixture-amount experiment

**Figure 2:**
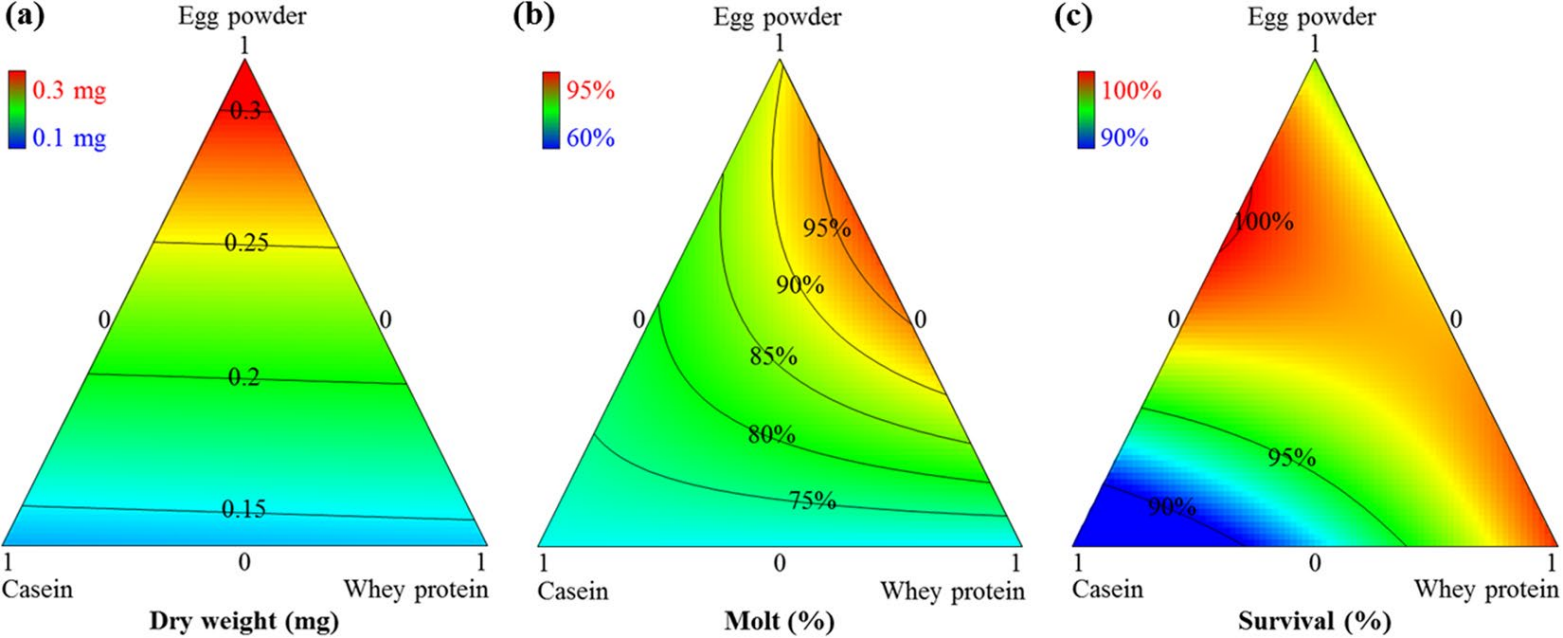
Ternary plots of larval responses for WCR reared on different diets from mixture-amount design of casein: egg powder: whey protein at 10 days post infestation. (**a**) weight, (**b**) survival, and (**c**) molting. Color bars display the magnitude of the measured response. Total amount of mixture = 3 grams.

**Figure 3:**
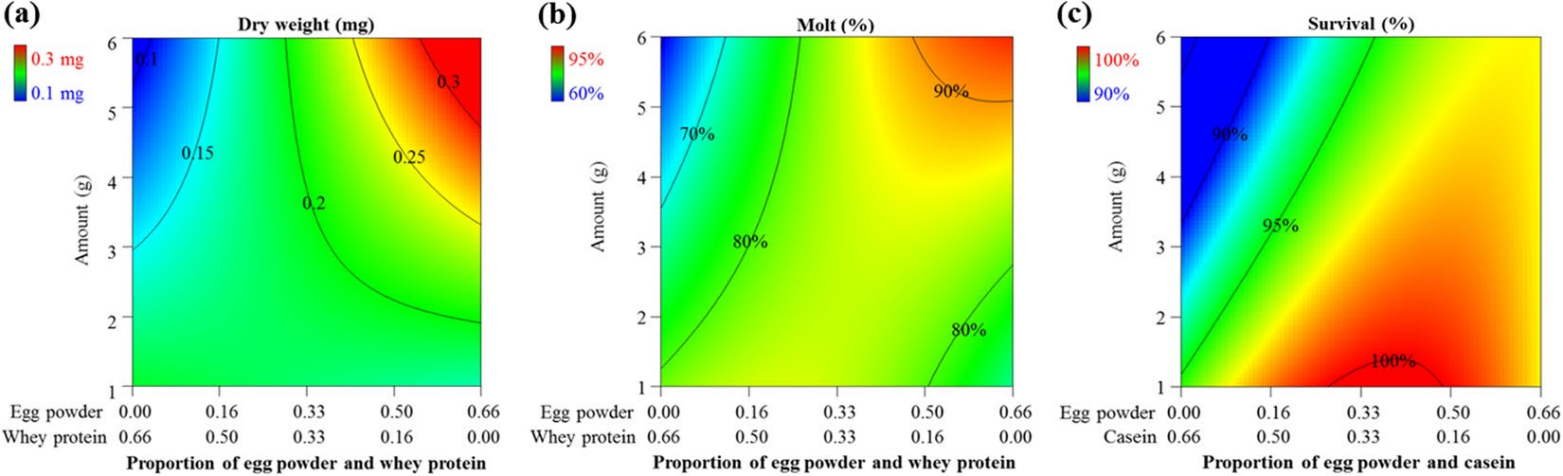
Contour plots of the interaction of egg powder, casein and concentration on WCR larvae reared on different diets from the mixture-amount design at 10 days post infestation. (**a**) weight, (**b**) survival, and (**c**) molting. (**a**,**b**) Proportions of casein = 0.34; (**c**) Proportions of whey protein = 0.34. Color bars display the magnitude of the measured response.

The three-protein mixture-amount (casein, egg powder, and whey protein) yielded significant response surface models for all three measures, including larval weight (*p < *0.0001, *F*_*4*,*26*_ = 12.48) with significant LOF (*p < *0.0001), molting (*p = *0.0006, *F*_*5*,*25*_ = 6.44) with significant LOF (*p = *0.0011), and survival (*p < *0.0001, *F*_*4*,*26*_ = 23.26) with insignificant LOF (*p = *0.0584) (see Supplementary Table S5). Models for weight and molting had significant LOFs due to very small values of pure error. Sums of square of pure error for models for weight and molting were 0.0013 and 0.0110, respectively. The R-squared, predicted R-squared and adjusted R-squared values of all models were clustered in within reasonable agreement (difference <0.2). Ternary plots showed the relationships between protein sources, mixture amount and larval performance by estimating the influences of all possible combinations of mixture components on measured variables (Figs 2 and 3). Color and labelled isobars indicated the magnitude of the response variables in a dimension that can be envisioned as perpendicular to the page.

All response models indicated that egg powder was the most important component to maximize all development traits (Fig. 2a–c). High proportions of egg powder resulted in an increase in all measures of larval performance whereas casein at high proportions resulted in a decrease in all response measures, especially in survival. An increase in the proportion of whey protein resulted in an increase in survival, but whey protein at high proportions had negative effects on weight and molting. There was a synergistic blending effect of egg powder and whey protein on larval molting, indicating an optimal two-component axial blend for egg powder: whey protein at a ratio of 3:2 (see Supplementary Fig. S1).

The contour plots showed interactive effects between egg powder and whey protein and egg powder and casein in relation to varying total amount mixture while proportions of other components (casein or whey protein) were held constant (Fig. 3a–c). The results showed that egg powder at high proportions with higher total amount of mixtures could yield better larval weight, molting, and survival. Since egg powder at high proportions had >95% survival, the minor negative effect of increasing the proportion of egg powder at higher total amount of diet on survival was not considered.

### Egg powder optimization

**Figure 4:**
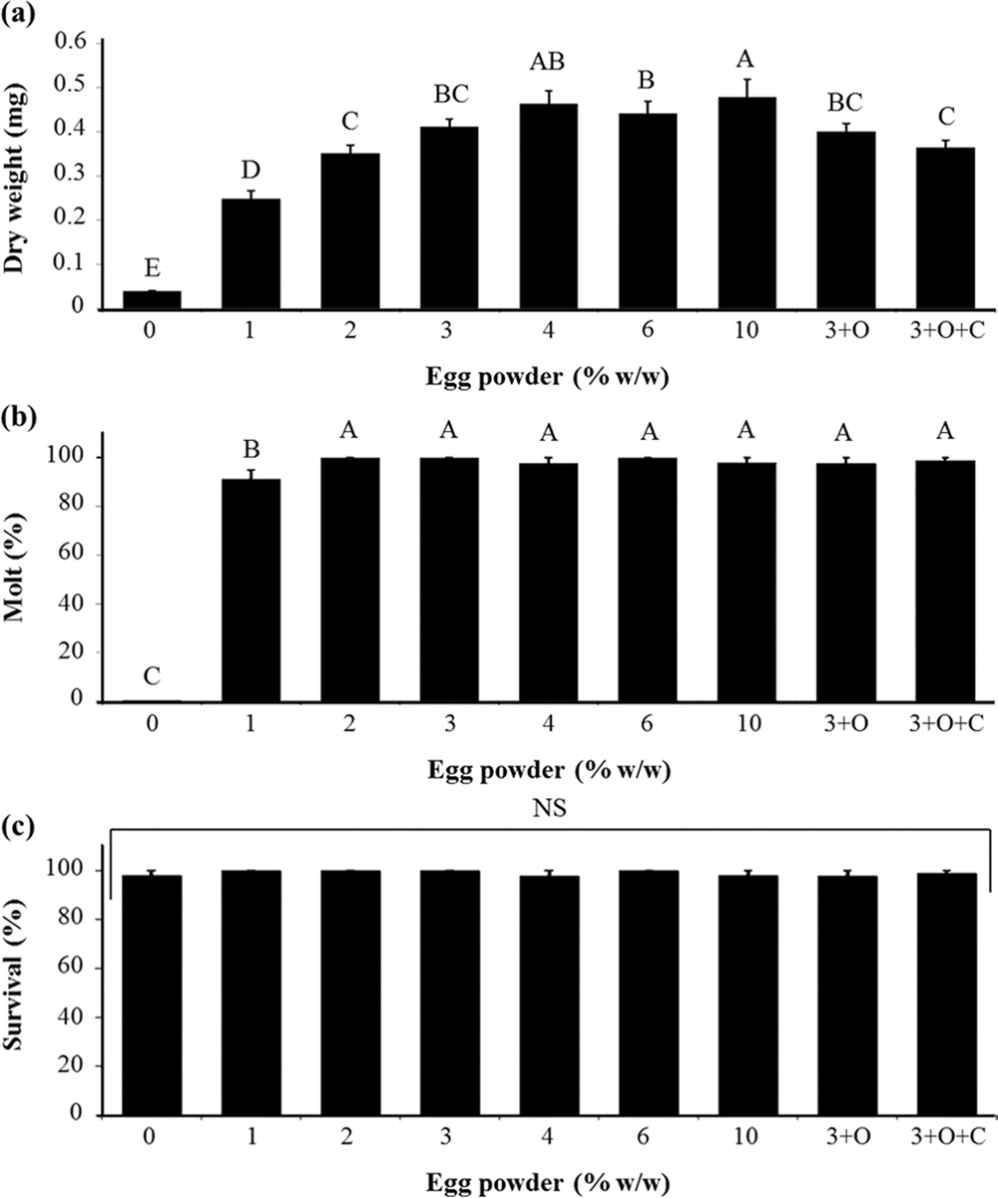
Larval dry weight, survival, and percent successful completion of molt for western corn rootworm larvae reared on diets containing egg powder at different concentrations. 3 + O: diets contain 3% egg powder plus linseed oils and wheat germ oils, 3 + O + C: diets contain 3% egg powder plus lipid components and corn root powder. (a) weight (*p* < 0.0001, *F*_8,32_ = 32.95), (**b**) molting (*p* < 0.0001, *F*_8,32_ = 97.73), and (**c**) survival (*p* = 0.8155, *F*_8,32_ = 0.54). Means with bars followed by different letters are significantly different (*p* < 0.05). Means ± SEM.

**Figure 5:**
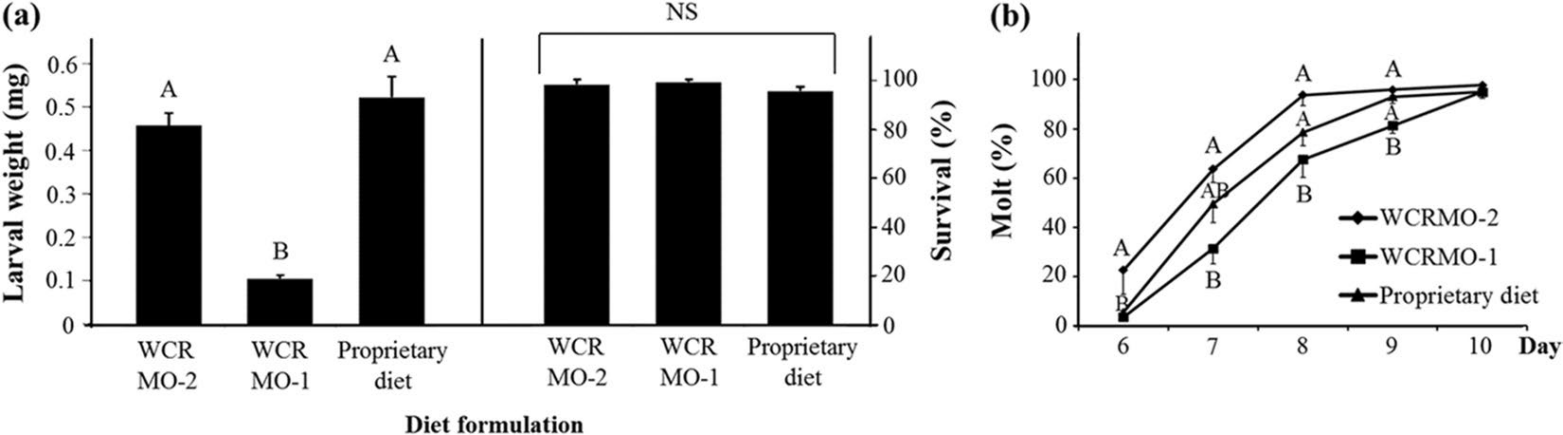
Larval dry weight, survival and percent successful completion of molt for western corn rootworm larvae from a diapausing strain reared on WCRMO-2, WCRMO-1, and a proprietary diet (Diet D from Meihls *et al*.^24^) for 10 days. Means with bars followed by different letters are significantly different. Statistics: weight (*p* < 0.0001, *F*_2,10_ = 79.85), survival (*p* = 0.2538, *F*_2,10_ = 1.58), molting: 6 days (*p* = 0.0076, *F*_2,17_ = 6.59), 7 days (*p* = 0.0081, *F*_2,17_ = 6.47), 8 days (*p* = 0.0146, *F*_2,17_ = 5.48), 9 days (*p* = 0.0336, *F*_2,17_ = 4.17), 10 days (*p* = 0.6767, *F*_2,17_ = 0.40). Means ± SEM.

Increases in egg powder up to a proportion of 4% (w/w) and 2% (w/w) resulted in positive larval weight gain (Fig. 4a) and molting (Fig. 4b), respectively, whereas additions of egg powder had no significant effect on survival (Fig. 4c). All diets tested had larval survivorship to 10 d that were higher than 95%, and there were no significant differences in survival. Additions of oil components alone and oil components plus corn root powder to the diet did not yield a better formulation for any developmental traits (Fig. 5a–c).

**Figure 6:**
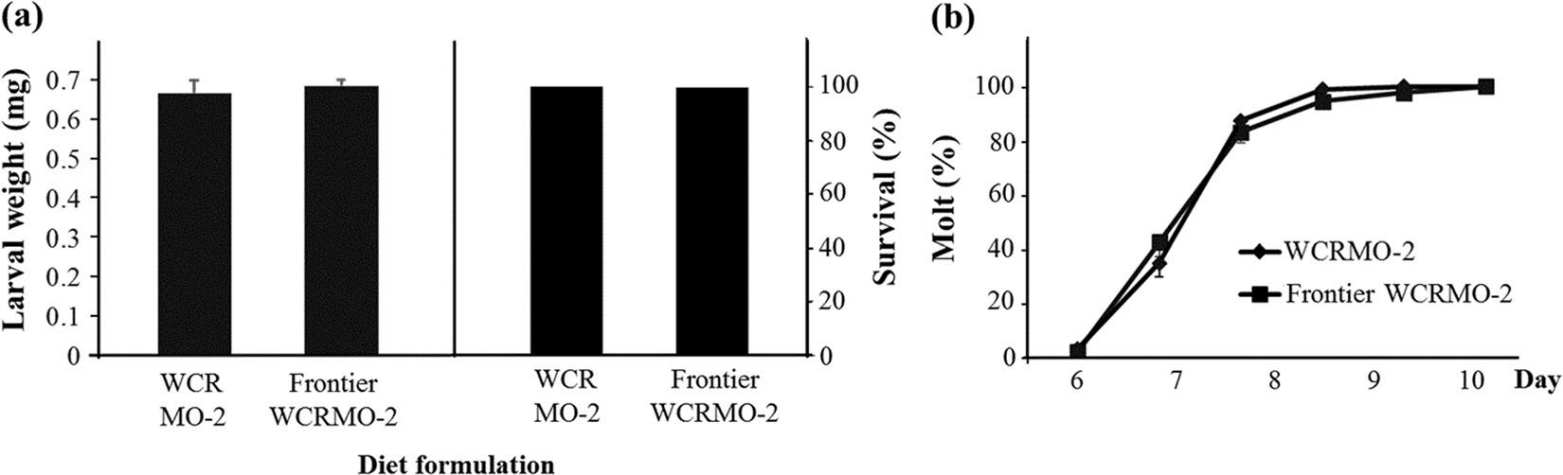
Larval dry weight, survival and percent successful completion of molt for western corn rootworm larvae from a non-diapausing strain reared on WCRMO-2 and Frontier WCRMO-2 for 10 days. Means ± SEM.

The egg powder optimization produced a superior formulation, referred to hereafter as WCRMO-2 (Table 1). Compared with previous published WCR diets^18,19^, WCRMO-2 had no corn root powder, additions of egg powder and glucose, and removal of oil components, casein and sucrose. At 10 days post infestation, larval dry weight increased 4 fold with WCRMO-2 as compared to WCRMO-1 and survival and molting rate of WCR larvae reared on WCRMO-2 were approximately 97%. Additionally, there was no significant difference in all measured developmental traits when WCR larvae were reared on WCRMO-2 or the current best proprietary diet (Fig. 5). A commercial version of WCRMO-2, referred to hereafter as Frontier WCRMO-2, was also created through the collaboration with Frontier Scientific Services (Newark, DE, USA). There was no significant difference between WCRMO-2 and Frontier WCRMO-2 in all life history parameters measured (Fig. 6).

**Table 1:** Artificial diets for WCR larvae (106 g).

Ingredients	Pleau diet^a^	WCRMO-1^b^	WCRMO-2
Egg powder	—	—	4.0 g
Glucose	—	—	1.0 g
Wheat germ (raw, ground)	5.45 g	5.5 g	6.0 g
Cellulose	1.38 g	1.5 g	1.0 g
Agar	1.45 g	1.5 g	1.5 g
Casein	3.23 g	2.5 g	—
Corn root powder	0.63 g	1.5 g	—
Sucrose	3.85 g	2.5 g	—
Linseed oil, raw	40 µl	25 µl	—
Wheat germ oil	—	25 µl	—
Cholesterol	6 mg	6 mg	6 mg
Wesson’s salt mix	0.93 g	0.93 g	0.93 g
Vanderzant Vitamin mix	0.9 g	0.9 g	0.9 g
Methyl paraben	0.1 g	0.1 g	0.1 g
Sorbic acid	64 mg	64 mg	64 mg
Potassium hydroxide (10%)	3.5 ml	3.5 ml	2.75 ml
Streptomycin (12.8 mg/ml)	6.4 mg	6.4 mg	6.4 mg
Chlortetracycline (10.0 mg/ml)	6.4 mg	6.4 mg	6.4 mg
Distilled water	88 ml	88 ml	88 ml
Green food coloring	64 µl	64 µl	64 µl

^a^Pleau *et al*.^19^, ^b^Huynh *et al*.^18^. ‘—‘: absence.

### Contamination

Low rates of contamination on diet bioassays have been achieved by careful attention to clean laboratory practices^18,23,24^. In this report observed contamination of all experiments was less than 1%.

## Discussion

Current published WCR diets require corn root powder as a key component because of its positive influence on larval performance^18,19^. However, corn root powder is the only ingredient that is not currently commercially available and makes the WCR diet unavailable for many users. We applied response surface methodology combined with n-dimensional mixture designs to develop an improved diet for WCR without corn root powder by systematically exploring the effects of different protein sources on WCR based on life history parameters (weight, molting, and survival). By using geometric and mathematical approaches, we evaluated the influence of eight different protein sources to identify key protein sources and characterized and maximized benefits of the key protein, resulting in an improved formulation that will be more accessible to users.

The eight-protein mixture experiment indicated that animal protein sources supported all larval developmental traits (weight, molting, and survival) better than other protein sources such as plant and yeast proteins (Fig. 1). All three animal proteins had the greatest positive effects on overall larval performance. Increasing proportions of three animal proteins (casein, egg powder, and whey protein) resulted in greater improvements in overall response measures compared with other tested proteins. For plant protein sources, additions of plant protein and cottonseed meal improved all measured responses, but increases in the proportions of corn gluten meal and perfect amino had detrimental effects on the three measured responses. Compared with other proteins tested, yeast protein had the smallest effects on all developmental traits. Pleau *et al*.^19^ reported a similar pattern in which WCR larvae performed better with proteins (casein and sodium caseinate) derived from animals compared with proteins (lima bean and pinto beans) derived from plants. Research with lepidopteran insects has also documented similar results. The African cotton leafworm, *Spodoptera littoralis* (Boisduval), grew better on an artificial diet that contained casein as its protein supplement compared to those reared on a diet formulation that casein was substituted with zein, a maize-derived protein^34^. Damodaran and Parkin^35^ documented that nutritional quality in animal proteins are better than that of plant proteins. Animal proteins (e.g., egg yolk, casein) usually provide a complete and balanced level of essential amino acids, while the essential amino acids in proteins of plants (e.g., cereals, legumes) are often not fully presented and are usually not presented at the well-balanced proportions. Bioavailability of proteins of animal origin are better than those of plant origin as well^35^.

The three-protein mixture-amount experiment to characterize the key protein components revealed that egg powder was the best protein source and egg powder alone at higher amount of diet blends could yield the best formulation based on all developmental traits (weight, molt, and survival) compared to possible combinations of three animal proteins, i.e. casein, egg powder, whey protein (Figs 2 and 3). There was a nonlinear synergy blending effect between egg powder and whey protein on molting (see Supplementary Fig. S1) and this interaction was important when egg powder was at low proportions (Fig. 3b). Casein at high proportions had a negative effect on survival and molting. The similar pattern of the negative effects of casein on larval survival and molting was reported in previous work of WCR diets^18,19^. These findings suggest that egg powder was the single best protein source. Egg powder used in the current study is derived from whole eggs and therefore is itself a complex mixture that mainly contains proteins but also additional nutritional components (e.g., lipids, vitamins, minerals). Further efforts to identify which components in egg powder are driving the effect would require fractionating egg powder into its components followed by a series of multivariate experiments.

The application of mixture experiments with response surface modeling has shown value in insect diet improvement by identifying and maximizing key ingredients in existing coleopteran diets^17,18,25–27^. By using this approach, we characterized eight different protein ingredients from animal, plant and yeast sources and maximized the key protein component based on all larval response measures. This resulted in a superior formulation (WCRMO-2) with the addition of egg powder, removal of corn root powder, removal of lipid components (linseed oils and wheat germ oils) and a substitution of sucrose with glucose compared with WCRMO-1^18^. At 10 days post infestation, the level of diet improvement included a 4-fold increase in larval weight gain with WCRMO-2 as compared to WCRMO-1 whereas survival and molting rate of WCR larvae on WCRMO-2 at approximately 97%. There was no significant difference on overall larval performance when WCR larvae were reared on WCRMO-2 compared with the current superior proprietary diet (Fig. 5). Additionally, WCRMO-2 without corn root powder had very low levels of contamination. The new formulation significantly improved larval performance and is more widely available for users. This is a significant accomplishment toward the long-term goal to develop the ideal diet that is publicly available, easy to use, and produces insects that are physically similar to WCR larvae fed on corn roots. For the relevant comparison, larval development when reared on WCRMO-2 slightly lags behind those reared on corn roots. The developmental times of 1^st^ instar of WCR on WCRMO-2 at 25 °C averaged 6.1 ± 0.07 d, whereas that of WCR on corn at 24 °C were 4.8 ± 0.09 d for male and 5.3 ± 0.17 d for female^36^. Further efforts are ongoing to develop an efficiency system for continuous rearing WCR on WCRMO-2 in multiple generations.

Artificial diets for WCR larvae are used in diet assays to detect the susceptibility of WCR populations to Bt toxins and other sources. Currently, each of the major maize seed companies have developed their own proprietary diet formulations and evaluated their own proteins on their own diets. There is considerable variation among proprietary diets and published diets currently in use for rearing rootworm larvae^24^. Evaluating rootworm toxins on differing diets does not allow direct comparisons between proteins due to differences in artificial diet formulations. In fact, nutrition may have a significant effect on the toxicity of Bt in WCR as has been shown for lepidopteran species^37–41^. In the cabbage looper, *Trichoplusia ni* Hübner, previously unexposed insects fed Cry1Ac incorporated diet were less susceptible on diets containing a low protein:carbohydrate (35:65) ratio than diet containing a high protein: carbohydrate ratio (90:10)^41^. If nutrition similarly affects coleopteran response to toxins, then differences in the artificial diets between individual companies will complicate direct comparisons between assays; and consequently may provide an inaccurate phenotypic picture of the test populations as it relates to toxin susceptibility. A single optimized artificial diet is necessary to facilitate standardized corn rootworm resistance monitoring assays. All researchers, seed companies, and those performing EPA required monitoring should have access to the same diet formulation and preparation method. However, that level of diet standardization will only happen if the diet is commercially produced. We have collaborated with Frontier Scientific Services to establish a commercial version of WCRMO-2 (Frontier WCRMO-2) that is designed to service and be available for all researchers whether they are members of industry or university laboratories. We believe this formulation would facilitate resistance monitoring efforts and new research discoveries.

## Materials and Methods

### Insects

Eggs of WCR (primary diapausing and non-diapausing strains) were provided by the USDA-ARS laboratories in Brooking, SD and in Columbia, MO. The eggs were surface-sterilized using a procedure described in Pleau *et al*.^19^ (see Supplementary Methods).

### Diet preparation

The best proprietary WCR diet^24^ provided by industry was used within a week of receipt. Other diets were poured using a procedure described in Huynh *et al*.^18^ (see Supplementary Methods), except for a commercial version of WCRMO-2 (Frontier WCRMO-2, Frontier Scientific Services). A Frontier WCRMO-2 batch includes agar, a dry mix pack, and KOH solution. To make 1 L of Frontier WCRMO-2, agar (15.8 g) was added to 926 ml of cold water the solution was brought to a full boil for 1 minute while stirring regularly or until agar was completely melted. The agar solution was then transferred to a blender placed in a biological safety cabinet. When the agar solution had cooled to 65 °C, 147.4 g of the dry mix pack was added, and the mixture was blended for 1 minute or until mixed thoroughly. Subsequently, 26.3 ml of KOH solution were added and blended for 1 minute or until mix thoroughly. The diet solution was dispensed immediately into a 96-well plate using a repeater pipette (200 µl per well) or proper containers. The diet plate was opened to evaporate excess moisture in the biological cabinet for 10 min, stored in a refrigerator at 4 °C and used for assays within a week.

### Insect artificial diet bioassays

The diet bioassays were conducted as described by Huynh *et al*.^18^. All materials used in the diet assays were surface-treated via exposure to UV light for 10 min in biological cabinet. Each formulation was randomly assigned to a 12-well row of the 96-well plate and replicated 5 times for a total of 60 larvae per each formulation. Each well was infested with one larva, which hatched within 24 h, using a fine paintbrush. The plate was covered with a sealing film (TSS-RTQ-100, Excel Scientific, Inc., Victorville, CA, USA). For ventilation, a hole was made with a number zero insect pin in the sealing film over each well. The plates were kept in an incubator (03009412, Sheldon Manufacturing Inc., Cornelius, OR, USA) at 25 °C in darkness for 10 days. Larval weight, molting, survival, and evidence of diet contamination were recorded at 10 days. For larval dry weight, all live larvae were pooled per replicate (12 possible) into 95% ethanol, dried in an oven (602752, Blue M Therm Dry Bacteriological Incubator) at 50 °C for 2 days, and weighed using a micro balance (MSU6.6S-000-DM, Sartorius Lab Instruments GmbH & Co. KG, Goettingen, Germany).

### Eight-protein screening design for substitutes for corn root powder

Previous work by Pleau *et al*.^19^ and Huynh *et al*.^18^ illustrated that protein components were key ingredients that had positive effects on overall WCR larval performance. To eliminate corn root powder, we explored contributions of eight different protein sources (i.e., corn gluten meal, cottonseed, casein, egg powder, plant protein, perfect amino, yeast extract, and whey protein) to three life history parameters (weight, molt, and survival) by constructing an eight-component I-optimal mixture design sufficient to satisfy a Scheffé quadratic polynomial response surface model^30^. I-optimal mixture designs refer to a mathematical algorithm that identifies diet blends that predict precise responses by minimizing the average variance of prediction across the design space^42,43^. Additional points to estimate lack of fit, which measures how well the model is fitted by the data, and replicated points to obtain sufficient degree of freedom for estimating pure error and to attain a uniform leverage for design spaces^44^ were also generated. This design was generated with Design-Expert^®^ (Stat-Ease, Inc., Minneapolis, MN, USA) that had 30 design points with 7 model, 17 lack of fit and 5 pure error degrees of freedom (see Supplementary Table S1). Other ingredients were kept constant (see Supplementary Table S2).

### Three-protein mixture-amount design for characterizing key protein sources

Based on the results of the first experiment, three proteins that had the greatest effects on WCR larval performance (weight, molt, and survival) were used to construct a D-optimal mixture-amount design sufficient for modeling a Scheffé quadratic-quadratic polynomial response surface model^30^. D-optimal mixture designs refer to the mathematical algorithm that chooses diet blends that focus on building precise model estimation^43,45^ by maximizing the determinant of the information matrix, and are appropriate for experimental designs that combine mixture and process variables^46^. A mixture-amount experiment is a type of mixture experiment that varies both mixture component proportions and the total amount of the mixture^30^. In this experiment, a three-component mixture design was generated with Design-Expert^®^ (Stat-Ease, Inc.) to characterize the effects of varying three animal proteins (casein, egg powder, and whey protein) from 0 to 1. The amounts of these proteins were varied in blends from 1 gram to 6 grams. This design consisted of 32 design points with 17 model, 6 lack of fit and 8 pure error degrees of freedom (see Supplementary Table S3). Additional design points and replicated points were included in order to satisfy the model terms, estimate lack of fit, attain sufficient degrees of freedom for estimating pure error, and to attain a uniform leverage for design spaces^44^. Other ingredients were kept constant (see Supplementary Table S2).

### Egg powder experiment to identify optimum formulation

The mixture-amount experiment revealed that egg powder alone had the largest effect on larval performance. An experiment to find the optimum amount of egg powder was conducted by varying the concentration of egg powder (i.e., 0%, 1%, 2%, 3%, 4%, 6%, and 10% w/w). Additionally, formulations containing 3% egg powder with additions of lipid components (linseed oils and wheat germ oils) and lipid components plus corn root powder were tested. The amount of lipid components and corn root powder added were the same as in WCRMO-1 diet^18^. The proportions of water were changed to incorporate changes in egg powder proportions and additions of lipid components and corn root powder. Other ingredients were kept constant (see Supplementary Table S2). Two WCR diets including WCRMO-1^18^ and the current best proprietary diet^24^ were also included as positive controls.

### Statistical analyses

The percentage of larvae surviving and molting was determined by dividing the number of larvae surviving and molting by the number of larvae initially infested and multiplying by 100. Weight per larva was determined by dividing the dry weight by the number of larvae that survived.

In the eight-protein mixture and three-protein mixture-amount experiments, the best fit model for each measured response (weight, molting, and survival) was selected from all possible models from linear to quartic polynomials generated with Design Expert^®^ (Stat-Ease, Inc.). Model selection was based on several criteria including low model *P*-value, lack of fit *P*-value, low standard deviation, high R-values (R-squared, adjusted R-squared, and predicted R-squared)^26^, close agreement between adjusted R-squared and predicted R-squared, and a low PRESS value^47,48^. Once more than one satisfactory model was generated, adequacy tests were performed to further evaluate the selected model as described by Anderson and Whitcomb^49,50^.

In the egg powder experiment, all response measures (weight, molting, and survival) were analyzed as a randomized complete block design using PROC MIXED in SAS^51^.

## Supplementary information

Supplementary information

## Data Availability

All pertinent data are found in the figures and tables. Requests for data and additional information should be submitted to the corresponding author.

## References

[1] Mitchell, P. D. Costs and benefits of controlling pest *Diabrotica* in maize in the United States. In, 24^th^ IWGO Conference and 3^rd^ International Conference of *Diabrotica* Genetics. IWGO, Freiburg, Germany (2011).

[2] Spike BP, Tollefson JJ (1989). Relationship of plant phenology to corn yield loss resulting from western corn rootworm (Coleoptera: Chrysomelidae) larval injury, nitrogen deficiency, and high plant density. J. Econ. Entomol..

[3] Spike BP, Tollefson JJ (1991). Yield response of corn subjected to western corn root worm (Coleoptera: Chrysomelidae) infestation and lodging. J. Econ. Entomol..

[4] Kahler AL, Olness AE, Sutter GR, Dybing CD, Devine OJ (1985). Root damage by western corn rootworm and nutrient content in maize. Agron. J..

[5] Palmer LT, Kommedahl T (1969). Root-infecting *Fusarium* species in relation to rootworm infestations in corn. Phytopathology.

[6] Kurtz B, Karlovsky P, Vidal S (2010). Interaction between western corn rootworm (Coleoptera: Chrysomelidae) larvae and root-infecting *Fusarium verticillioides*. Environ. Entomol..

[7] Spike BP, Tollefson JJ (1988). Western corn rootworm (Coleoptera: Chrysomelidae) larval survival and damage potential to corn subjected to nitrogen and plant density treatments. J. Econ. Entomol..

[8] Ball HJ, Weekman GT (1963). Differential resistance of corn rootworms to insecticides in Nehraska and adjoining States. J. Econ. Entomol..

[9] Meinke LJ, Siegfried BD, Wright RJ, Chandler LD (1998). Adult susceptibility of Nebraska western corn rootworm (Coleoptera: Chrysomelidae) populations to selected insecticides. J. Econ. Entomol..

[10] Pereira AE (2015). Evidence of field-evolved resistance to bifenthrin in western corn rootworm (*Diabrotica virgifera virgifera* LeConte) populations in western Nebraska and Kansas. PloS one.

[11] Levine E, Spencer JL, Isard SA, Onstad DW, Gray ME (2002). Adaptation of the western corn rootworm to crop rotation: evolution of a new strain in response to a management practice. Am. Entomol..

[12] Gray ME, Sappington TW, Miller NJ, Moeser J, Bohn MO (2009). Adaptation and invasiveness of western corn rootworm: intensifying research on a worsening pest. Ann. Rev. Entomol..

[13] Gassmann AJ, Petzold-Maxwell JL, Keweshan RS, Dunbar MW (2011). Field-evolved resistance to Bt maize by western corn rootworm. PloS one.

[14] Zukoff SN (2016). Multiple assays indicate varying levels of cross resistance in Cry3Bb1-selected field populations of the western corn rootworm to mCry3A, eCry3.1Ab, and Cry34/35Ab1. J. Econ. Entomol..

[15] Ludwick DC (2017). Minnesota field population of western corn rootworm (Coleoptera: Chrysomelidae) shows incomplete resistance to Cry34Ab1/Cry35Ab1 and Cry3Bb1. J. Appl. Entomol..

[16] EPA. United States Environmental Protection Agency docket for corn rootworm resistance management and framework for Bt corn. (United States Environmental Protection Agency, Washington, DC, United States of America, 2016).

[17] Lapointe SL, Evens TJ, Niedz RP, Hall DG (2010). Artificial diet optimized to produce normative adults of *Diaprepes abbreviatus* (Coleoptera: Curculionidae). Environ. Entomol..

[18] Huynh MP (2017). Diet improvement for western corn rootworm (Coleoptera: Chrysomelidae) larvae. PloS one.

[19] Pleau MJ, Huesing JE, Head GP, Feir DJ (2002). Development of an artificial diet for the western corn rootworm. Entomol. Exp. Appl..

[20] Sutter GR, Krysan JL, Guss PL (1971). Rearing the southern corn rootworm on artificial diet. J. Econ. Entomol..

[21] Marrone PG, Ferri FD, Mosley TR, Meinke LJ (1985). Improvements in laboratory rearing of the southern corn rootworm, *Diabrotica undecimpuncta howardi* Barber (Coleoptera: Chrysomelidae), on an artificial diet and corn. J. Econ. Entomol..

[22] Rose RI, McCabe JM (1973). Laboratory rearing techniques for the southern corn rootworm. J. Econ. Entomol..

[23] Ludwick DC (2018). A new artificial diet for western corn rootworm larvae is compatible with and detects resistance to all current Bt toxins. Sci. Rep..

[24] Meihls, L. N. *et al*. Comparison of six artificial diets for western corn rootworm bioassays and rearing. *J*. *Econ*. *Entomol*. **toy268** (2018). 10.1093/jee/toy268 30189100

[25] Huynh MP (2019). Multidimensional approach to formulating a specialized diet for northern corn rootworm larvae. Sci. Rep..

[26] Lapointe SL, Evens TJ, Niedz RP (2008). Insect diets as mixtures: Optimization for a polyphagous weevil. J. Ins. Physiol..

[27] Lapointe SL, Niedz RP, Evens TJ (2010). An artificial diet for *Diaprepes abbreviatus* (Coleoptera: Curculionidae) optimized for larval survival. Fla. Entomol..

[28] Scheffé H (1958). Experiments with mixtures. Royal Stat. Soc., Series B (Methodological).

[29] Piepel GF, Cornell JA (1987). Designs for mixture-amount experiments. J. Qual. Tech..

[30] Cornell, J. A. *Experiments with mixtures: designs*, *models*, *and the analysis of mixture data*, *3rd ed*. (John Wiley & Sons, Inc., New York, USA, 2002).

[31] Myers, R. H., Montgomery, D. C. & Anderson-Cook, C. M. *Response surface methodology: process and product optimization using designed experiments*, *4th ed*., (John Wiley & Sons, 2016).

[32] Huynh MP (2019). Characterization of corn root factors to improve artificial diet for western corn rootworm (Coleoptera: Chrysomelidae) larvae. J. Insect Sci..

[33] Smith, W. F. *Experimental design for formulation*. (SIAM, Philadelphia, PA, USA, 2005).

[34] Lee K, Simpson S, Wilson K (2008). Dietary protein‐quality influences melanization and immune function in an insect. Funct. Ecol..

[35] Damodaran, S. & Parkin, K. L. *Fennema’s food chemistry*. (CRC press, Boca Raton, FL, USA, 2017).

[36] Jackson JJ, Elliott NC (1988). Temperature-dependent development of immature stages of the western corn rootworm, *Diabrotica virgifera virgifera* (Coleoptera: Chrysomelidae). Environ Entomol..

[37] Janmaat AF, Myers JH (2005). The cost of resistance to *Bacillus thuringiensis* varies with the host plant of *Trichoplusia ni*. Proc. Royal Soc. Lond.

[38] Bird LJ, Akhurst RJ (2007). Effects of host plant species on fitness costs of Bt resistance in *Helicoverpa armigera* (Lepidoptera: Noctuidae). Biol. Control.

[39] Raymond B, Sayyed AH, Wright DJ (2006). Host plant and population determine the fitness costs of resistance to *Bacillus thuringiensis*. Biol. Lett.

[40] Blanco CA (2009). Response of *Heliothis virescens* (Lepidoptera: Noctuidae) strains to *Bacillus thuringiensis* Cry1Ac incorporated into different insect artificial diets. J. Econ. Entomol..

[41] Orpet RJ (2015). Effects of dietary protein to carbohydrate ratio on Bt toxicity and fitness costs of resistance in *Helicoverpa zea*. Entomol. Exp. et Appl..

[42] Laake, P. On the optimal allocation of observations in experiments with mixtures. *Scand*. *J*. *Stat*, 153–157 (1975).

[43] Goos P, Jones B, Syafitri U (2016). I-optimal design of mixture experiments. J. Am. Stat. Assoc..

[44] Weisberg, S. *Applied linear regression*, *2nd ed*., (Wiley & Sons, 1985).

[45] Czitrom V (1988). Mixture experiments with process variables: D-optimal orthogonal experimental designs. Commun. Stat. Theory Methods.

[46] Eriksson, L., Johansson, E., Kettaneh-Wold, N., Wikström, C. & Wold, S. Design of experiments. *Principles and Applications*, *Learn ways AB*, *Stockholm* (2000).

[47] Myers, R. H. & Montgomery, D. C. *Response surface methodology: process and product optimization using designed experiments*, *2nd ed*. (Willey, 2002).

[48] Allen DM (1971). Mean square error of prediction as a criterion for selecting variables. Technometrics.

[49] Anderson, M. J. & Whitcomb, P. J. *RSM simplified: optimizing processes using response surface methods for design of experiments*. (CRC press, 2004).

[50] Anderson MJ, Whitcomb PJ (2007). Using graphical diagnostics to deal with bad data. Qual. Eng..

[51] SAS. SAS version 9.4. (SAS Institute, Cary, N.C, USA, 2013).

