# Supplementary material for: Development of an improved and accessible diet for western corn rootworm larvae using response surface modeling
Source: Sci Rep. 2019 Nov 5;9:16009. doi: 10.1038/s41598-019-52484-z (PMC6831680; doi:10.1038/s41598-019-52484-z)
Supplement: Supplementary file 1 — Supplementary information [file 41598_2019_52484_MOESM1_ESM.docx]

**Supplementary Information**

**Development of an improved and accessible diet for western corn rootworm larvae using response surface modeling**

Man P. Huynh^1*^, Bruce E. Hibbard^2^, Michael Vella^3^, Stephen L. Lapointe^4^, Randall P. Niedz^4^, Kent S. Shelby^5^, Thomas A. Coudron^5^

^1^Division of Plant Sciences, University of Missouri, Columbia, Missouri, 65211, USA

^2^Plant Genetics Research Unit, USDA-Agricultural Research Service, Columbia, Missouri, 65211, USA

^3^Frontier Scientific Services, Newark, Delaware, 19711, USA

^4^United States Horticultural Research Laboratory, USDA-Agricultural Research Service, Fort Pierce, Florida, 34945, USA

^5^Biological Control of Insects Research Laboratory, USDA-Agricultural Research Service, Columbia, Missouri, 65203, USA

*Corresponding author: mphd32@mail.missouri.edu

**Supplementary Methods:**

**Insect egg sterilization.** The eggs were incubated in Petri dishes containing 70 mesh sieved-soil in an incubator at 25^o^C in complete darkness. When approximately 5% of the eggs hatched, the eggs were washed out of the soils using a 60 mesh sieve (Hogentogler & Co. Inc., Columbia, MD) with 25^o^C water. The remaining eggs were surface-treated based on descriptions by Pleau et al.^1^. First, the eggs were submerged in undiluted Lysol^®^ (Reckitt Benckiser, LLC, Parsippany, NJ) for 3 minutes and then the supernatant was removed and the eggs were triple rinsed with distilled water. Next, the eggs were submerged in 10% formalin (HT501128, Sigma Aldrich, St. Louis, MO) for 3 minutes, and then triple rinsed with distilled water after removing the supernatant again. Finally, the eggs were dispensed onto a coffee filter paper (Pure Brew, Rockline Industries, Sheboygan, WI) placed inside a 16 oz. cup (LG8RB-0090, Solo Cup Company, Lake Forest, IL) using a 1 ml disposable pipette (13-711-9a, Fisher Scientific, Pittsburg, PA). Several holes made in a lid (DM16R-0090, Solo Cup Company) of the container for ventilation with a number zero insect pin. The eggs were then incubated at 25^o^C in darkness. Neonate larvae that hatched in < 24 h were used for the insect bioassay.

**Diet preparation.**  Diets were made using a procedure described in Pleau et al.^1^, with some modifications by Huynh et al.^2^. Agar (A7002, Sigma-Aldrich) and distilled water were added to a 400 ml glass beaker and the solution was boiled for 2 minutes using a microwave. The molten agar solution was poured into a blender (Hamilton Beach, Inc., Model 51101BZ) placed in a biological safety cabinet (Nuaire, Biological safety cabinet). Next, dry diet ingredients, i.e. corn gluten meal (49760, Sigma-Aldrich), cottonseed meal (Down-To-Earth, Eugene, OR), plant protein (Perfect supplements, Coventry, RI), whey protein (ON, Downers Grove, IL), Perfect Amino® (Bodyhealth, Clearwater, FL), yeast extract (BP1422, Fisher Scientific, Fair Lawn, NJ), egg powder (Judee’s gluten free, Columbus, OH), casein (1100, Bio-Serv), wheat germ (1661, Bio-Serv, Flemington, NJ), cellulose (3425, Bio-Serv), glucose (D16, Fisher Scientific), sucrose (04821721, MP Biomedicals, Santa Ana, CA), corn root powder (USDA-ARS, Columbia, MO), salt mix (F8680, Bio-Serv), vitamin mix (V1007, Sigma-Aldrich), methyl paraben (H5501, Sigma-Aldrich), cholesterol (C8503, Sigma-Aldrich), and sorbic acid (S1626, Sigma-Aldrich) were added (if applicable) to the blender and mixed thoroughly at low speed. Bio-Serv has sold their insect diets to Frontier Agricultural Sciences and all Bio-Serv ingredients can be found at <https://insectrearing.com/product-category/diet-ingredients/>. The pH of the diet monitored by indicator strips (Whatman^®^ 09-876-18, GE Healthcare Bio-Sciences, Pittsburg, PA) was increased to a pH of 9.0 by adding 10% KOH (w/v) (P250, Fisher Scientific). Next, the diet was poured in to a 750 ml glass beaker placed on hot plate (Thermo scientific, Cimarec^TM^) at 65^o^C. A 96-well plate (3370, Corning Inc., Corning, NY) was used to accommodate the diet mixture with each well receiving 200 µl of the diet using a repeater pipette (Eppendorf repeater plus). The plate was opened to evaporate excess moisture for 15 minutes in the biological safety cabinet and then stored in a refrigerator at 4^ο^C. Diets were used within a week of being made for diet bioassays.

**Supplementary References**

1 Pleau, M. J., Huesing, J. E., Head, G. P. & Feir, D. J. Development of an artificial diet for the western corn rootworm. *Entomol. Exp. Appl.* **105**, 1-11 (2002).

2 Huynh, M. P. *et al.* Diet improvement for western corn rootworm (Coleoptera: Chrysomelidae) larvae. *PloS one* **12**, e0187997 (2017).

3 Cornell, J. A. Experiments with mixtures: designs, models, and the analysis of mixture data, 3rd ed. (John Wiley & Sons, Inc., New York, USA, 2002).

**Supplementary Table S1.** Diet blends of 8 proteins (gram) varied in a mixture screening design to rear western corn rootworm larvae

| Diet blend # | Corn gluten meal | Cottonseed meal | Casein | Egg powder | Plant protein | Perfect Amino | Yeast extract | Whey protein |
| --- | --- | --- | --- | --- | --- | --- | --- | --- |
| 1 | 0.38 | 0.38 | 0.38 | 0.38 | 0.37 | 0.37 | 0.37 | 0.37 |
| 2 | 0.43 | 0 | 0.43 | 0.43 | 0.43 | 0.43 | 0.43 | 0.42 |
| 3 | 0 | 0.43 | 0.43 | 0.43 | 0.43 | 0.43 | 0.43 | 0.42 |
| 4 | 0.43 | 0.43 | 0.43 | 0.43 | 0 | 0.43 | 0.43 | 0.42 |
| 5 | 0.43 | 0.43 | 0.43 | 0 | 0.43 | 0.43 | 0.43 | 0.42 |
| 6 | 0.38 | 0.38 | 0.38 | 0.38 | 0.37 | 0.37 | 0.37 | 0.37 |
| 7 | 0 | 0 | 0 | 0 | 0 | 0 | 3 | 0 |
| 8 | 0.43 | 0.43 | 0.43 | 0.43 | 0.43 | 0 | 0.43 | 0.42 |
| 9 | 0.38 | 0.38 | 0.38 | 0.38 | 0.37 | 0.37 | 0.37 | 0.37 |
| 10 | 3 | 0 | 0 | 0 | 0 | 0 | 0 | 0 |
| 11 | 0.38 | 0.38 | 0.38 | 0.38 | 0.37 | 0.37 | 0.37 | 0.37 |
| 12 | 0.19 | 0.19 | 0.19 | 0.19 | 0.19 | 1.67 | 0.19 | 0.19 |
| 13 | 0 | 0 | 0 | 3 | 0 | 0 | 0 | 0 |
| 14 | 0.19 | 0.19 | 0.19 | 1.67 | 0.19 | 0.19 | 0.19 | 0.19 |
| 15 | 0.43 | 0.43 | 0 | 0.43 | 0.43 | 0.43 | 0.43 | 0.42 |
| 16 | 1.67 | 0.19 | 0.19 | 0.19 | 0.19 | 0.19 | 0.19 | 0.19 |
| 17 | 0 | 0 | 3 | 0 | 0 | 0 | 0 | 0 |
| 18 | 0.19 | 0.19 | 1.67 | 0.19 | 0.19 | 0.19 | 0.19 | 0.19 |
| 19 | 0 | 0 | 0 | 0 | 0 | 0 | 0 | 3 |
| 20 | 0 | 0 | 0 | 0 | 0 | 3 | 0 | 0 |
| 21 | 0.19 | 1.67 | 0.19 | 0.19 | 0.19 | 0.19 | 0.19 | 0.19 |
| 22 | 0.19 | 0.19 | 0.19 | 0.19 | 0.19 | 0.19 | 0.19 | 1.67 |
| 23 | 0.43 | 0.43 | 0.43 | 0.43 | 0.43 | 0.43 | 0 | 0.42 |
| 24 | 0 | 0 | 0 | 0 | 3 | 0 | 0 | 0 |
| 25 | 0.19 | 0.19 | 0.19 | 0.19 | 1.67 | 0.19 | 0.19 | 0.19 |
| 26 | 0.19 | 0.19 | 0.19 | 0.19 | 0.19 | 0.19 | 1.67 | 0.19 |
| 27 | 0.38 | 0.38 | 0.38 | 0.38 | 0.37 | 0.37 | 0.37 | 0.37 |
| 28 | 0 | 3 | 0 | 0 | 0 | 0 | 0 | 0 |
| 29 | 0.43 | 0.43 | 0.43 | 0.43 | 0.43 | 0.43 | 0.42 | 0 |
| 30 | 3 | 0 | 0 | 0 | 0 | 0 | 0 | 0 |

**Supplementary Table 2.** Components that were held constant in diets used to rear western corn rootworm larvae in the 8-protein mixture experiment.

| **Constant components** |
| --- |
| 1. Agar |
| 1. Cellulose |
| 1. Chlortetracycline (10 mg/ml) |
| 1. Cholesterol |
| 1. Distilled water |
| 1. Food coloring |
| 1. Glucose |
| 1. Methyl paraben |
| 1. Potassium hydroxide (10%) |
| 1. Sorbic acid |
| 1. Streptomycin (12.8 mg/ml) |
| 1. Vanderzant vitamin mix |
| 1. Wesson's salt mix |
| 1. Wheat germ, ground |

**Supplementary Table 3.** Mixture-amount design points to determine the optimum combination of key protein ingredients. The proportions of each mixture component (casein, egg powder, and whey) that make up the total amount of protein in the diet (casein (g) + egg powder (g) + whey (g)) are shown.

| Diet blend # | Proportion of mixture components | | | Total mixture amount  [Casein + Egg powder + Whey protein] |
| --- | --- | --- | --- | --- |
|  | Casein | Egg powder | Whey protein |  |
| 1 | 0 | 0.5 | 0.5 | 6 |
| 2 | 0.16 | 0.16 | 0.67 | 2.25 |
| 3 | 0.16 | 0.67 | 0.16 | 2.25 |
| 4 | 0.5 | 0 | 0.5 | 6 |
| 5 | 1 | 0 | 0 | 3.5 |
| 6 | 0.5 | 0.5 | 0 | 6 |
| 7 | 1 | 0 | 0 | 6 |
| 8 | 0 | 1 | 0 | 6 |
| 9 | 1 | 0 | 0 | 1 |
| 10 | 0.5 | 0 | 0.5 | 1 |
| 11 | 0.67 | 0.16 | 0.16 | 4.75 |
| 12 | 0.16 | 0.16 | 0.67 | 4.75 |
| 13 | 0.5 | 0.5 | 0 | 1 |
| 14 | 0 | 1 | 0 | 6 |
| 15 | 0 | 1 | 0 | 3.5 |
| 16 | 0 | 0.5 | 0.5 | 1 |
| 17 | 1 | 0 | 0 | 1 |
| 18 | 0 | 0.5 | 0.5 | 3.5 |
| 19 | 0.5 | 0.5 | 0 | 3.5 |
| 20 | 0.5 | 0.5 | 0 | 1 |
| 21 | 0.5 | 0 | 0.5 | 3.5 |
| 22 | 0 | 0 | 1 | 1 |
| 23 | 0 | 0.5 | 0.5 | 1 |
| 24 | 0 | 0 | 1 | 1 |
| 25 | 0.16 | 0.67 | 0.16 | 4.75 |
| 26 | 0 | 1 | 0 | 1 |
| 27 | 0 | 0 | 1 | 3.5 |
| 28 | 0 | 0 | 1 | 6 |
| 29 | 1 | 0 | 0 | 6 |
| 30 | 0 | 1 | 0 | 1 |
| 31 | 0.67 | 0.16 | 0.16 | 2.25 |
| 32 | 0 | 0 | 1 | 6 |

**Supplementary Table 4.** *p*-values, regression coefficients and response surface model fitting diagnostic statistics for WCR larval responses to 8-component diet mixtures. A: corn gluten meal, B: cottonseed meal, C: casein, D: plant protein, E: whey protein, F: perfect amino, G: yeast extract, H: egg powder, ^a^Expressed in coded forms. Mixture component coding is L_Pseudo^3^.

|  | Weight  *p*-values | Regression coefficients^a^ | % Molt  *p*-values | Regression coefficients | % Survival  *p*-values | Regression coefficients |
| --- | --- | --- | --- | --- | --- | --- |
| Model | <0.0001 | - | <0.0001 | - | <0.0001 | - |
| Linear mixture | <0.0001 | - | <0.0001 | - | <0.0001 | - |
| A | - | 6.74 | - | -1.78 | - | 0.48 |
| B | - | 3.62 | - | -0.31 | - | 0.93 |
| C | - | 2.46 | - | -0.01 | - | 0.96 |
| D | - | -0.54 | - | 1.02 | - | 0.98 |
| E | - | 2.71 | - | -0.13 | - | 0.99 |
| F | - | 6.88 | - | -1.86 | - | 1.05 |
| G | - | 0.03 | - | 1.06 | - | 1.22 |
| H | - | 1.24 | - | 0.03 | - | 1.00 |
| A^2^ | - | - | 0.0082 | -1.26 | - | - |
| D^2^ | 0.0151 | 3.89 | 0.0058 | -1.39 | - | - |
| F^2^ | - | - | 0.0174 | -1.17 | 0.0026 | -0.48 |
| G^2^ | 0.0102 | 4.15 | 0.0002 | -2.12 | 0.0073 | -0.42 |
|  |  |  |  |  |  |  |
| Lack of fit | 0.5382 |  | 0.0397 |  | 0.0009 |  |
| Model type | Quadratic (reduced) |  | Quadratic (reduced) |  | Quadratic (reduced) |  |
| Transformation | Inverse square root |  | Base 10 log |  | Square root |  |
|  |  |  |  |  |  |  |
| R^2^ | 0.9500 |  | 0.9918 |  | 0.9581 |  |
| R^2^_adj_ | 0.9276 |  | 0.9868 |  | 0.9392 |  |
| R^2^_pred_ | 0.8196 |  | 0.9538 |  | 0.8243 |  |

**Supplementary Table 5.** *p*-values, regression coefficients and response surface model fitting diagnostic statistics for WCR larval responses to 3-protein mixture-amount experiment. A: egg powder, B: whey protein, C: casein. ^a^Expressed in coded forms, ^b^Concentration. Mixture component coding is L_Pseudo^3^.

|  | Weight  *p*-values | Regression coefficients^a^ | % Molt  *p*-values | Regression coefficients | % Survival  *p*-values | Regression coefficients |
| --- | --- | --- | --- | --- | --- | --- |
| Model | <0.0001 | - | 0.0006 | - | <0.0001 | - |
| Linear mixture | <0.0001 | - | 0.0119 | - | <0.0001 | - |
| A | - | 0.32 | - | 0.89 | - | 0.96 |
| B | - | 0.14 | - | 0.70 | - | 1.00 |
| C | - | 0.13 | - | 0.71 | - | 0.85 |
| A*B | - | - | 0.0267 | 0.65 | - | - |
| A*C | - | - | - | - | 0.0008 | 0.34 |
| B*C | - | - | - | - | - | - |
| A*Conc.^b^ | 0.0001 | 0.13 | 0.0127 | 0.16 | <0.0001 | -0.09 |
| B* Conc. | 0.0048 | -0.08 | 0.0039 | -0.16 | - | - |
|  |  |  |  |  |  |  |
| Lack of fit | <0.0001 |  | 0.0011 |  | 0.0584 |  |
| Model type | Linear mixture (reduced) x linear amount |  | Quadratic mixture (reduced) x linear amount |  | Quadratic mixture (reduced) x linear amount |  |
| Transformation | N/A |  | N/A |  | N/A |  |
|  |  |  |  |  |  |  |
| R^2^ | 0.6575 |  | 0.5629 |  | 0.7816 |  |
| R^2^_adj_ | 0.6048 |  | 0.4754 |  | 0.7480 |  |
| R^2^_pred_ | 0.5200 |  | 0.3775 |  | 0.6597 |  |

**
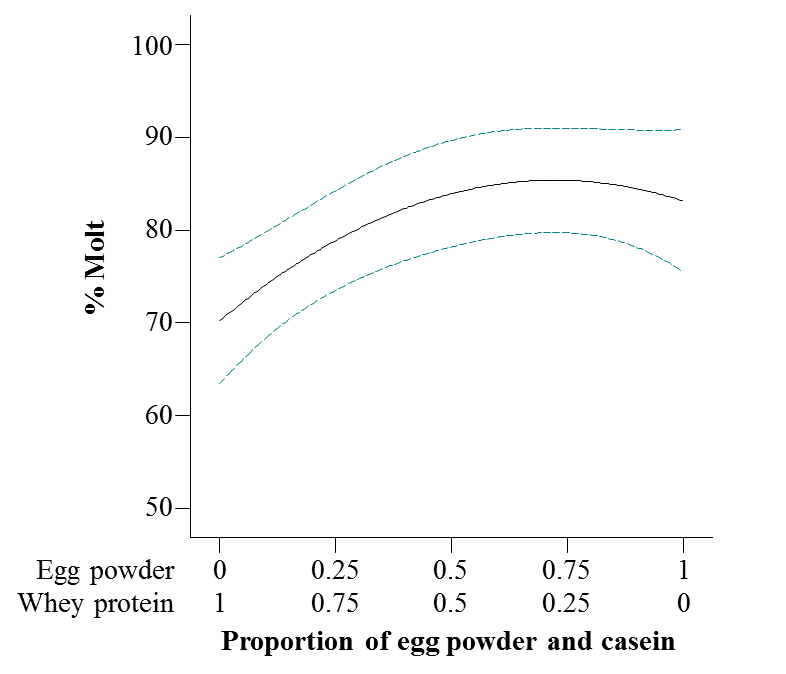
**

**Supplementary Figure 1.** Nonlinear blending effects of egg powder x whey protein on molting from the mixture-amount experiment. Dotted lines indicate 95% confidence interval bands.
